# Alphabet of Auscultation

**Published:** 1864-05

**Authors:** Tom. O. Edwards

**Affiliations:** Lancaster, Ohio


CHICAGO

MEDICAL JOURNAL.

VOL. XXI.] MAY, 1864. [No. 5.

### ORIGINAL COMMUNICATIONS.

### ALPHABET OF AUSCULTATION.

By TOM. O. EDWARDS, of Lancaster, Ohio.

The following short alphabet of auscultation has afforded me much advantage in studying diseases of the chest, and is I think, of sufficient interest to young beginners for publication.

*Two* dry sounds—Rhonchus and Sibilus.

*Two* moist sounds—Small and Large Crepitation.

*Three* vocal sounds—Bronchophany, Œgophany, and Pectoriloquy.

*Rhonchus*, or Snoring, found in large Bronchia, is produced by intumiscence or œdema of the mucous membranes of bronchii, on which phlegm impinges—found at the bifurcation of the bronchii. This sound indicates Bronchitis and is illustrated by the effused condition of the conjunctiva in conjunctivitis.

*Sibilus*, Wheezing, Whistling, or Cooing, is found in small Bronchia, and is as piccoli to bassoon—air drawn through the semi-closed, wet lips indicates this, while snoring illustrates Rhonchus.

*Small Crepitation* invariably attends pneumonia, and is the result of air passing through inflamed air-cells, clogged with sero sanguinolent secretions—rubbing the hair between the fingers illustrates the sound—also found in emplyrema and in a lung wounded from fractured ribs.

*Large Crepitation*, is like the breaking of large soap-bubbles, is found behind in lower lobes, in advanced bronchitis, and in third stage of pneumonia, and in emphysema, with œdema pulmonum.

*Bronchophany*, is increased resonance of voice, is produced by solidification of lungs, acting as a better conductor of sound than a healthy or vesicular portion. If from pneumonia, behind in lower lobes, if from tubercles, under clavicle.

*Œgophany*, or bleating of a goat, results from a small portion of affused lymph between the surfaces of the costal and pulmonary pleura, and is evidence of pleurisy—bad symptom in early pleurisy, good in advanced stage, showing effusion is being absorbed—found in lower lobes, behind the seat of pleurisy.

*Pectoriloquy*, the effect of the intonation of the voice passing up the stethoscope, as if it came directly from the chest. It is an unequivocal evidence of a cavity—generally in upper lobes, under the clavicle. It is simulated by placing the instrument on the wings of the thyroid cartilage and making the patient talk. These are the alphabet of auscultation, and however, imperfect, will greatly aid the beginner whose mind is apt to be filled with many indefinate phrases.

### AMERICAN MEDICAL ASSOCIATION.

The 15th Annual Meeting of the "American Medical Association" will be held in the City of New York, commencing on Tuesday, June 7th, 1864, at 10 o'clock A. M.

GUIDO FURMAN, M. D., Secretary.

New York, March, 1864.

